# Microbial load, carbon utilization potential, and immobilization efficiency vary across biochar types: implications for bioinoculant formulation

**DOI:** 10.3389/fmicb.2026.1869658

**Published:** 2026-09-01

**Authors:** Rashmi S. Dhanwar, Adarsh K. Singh, Munira Alateeqi, Rupali S. Thakur, Sanyogita Berde, Gaurav A. Nahar, Tushar D. Lodha, Andrew B. Ross, Ashvini Chauhan, Om Prakash

**Affiliations:** 1 Symbiosis Center for Climate Change and Sustainability, Symbiosis International (Deemed University), Pune, India; 2 School of Chemical and Process Engineering, University of Leeds, Leeds, United Kingdom; 3 Agharkar Research Institute, Pune, India; 4 Defiant Renewables Pvt Ltd, Pune, India; 5 Environmental Biotechnology School of the Environment, Florida A&M University, Tallahassee, FL, United States

**Keywords:** biochar, bioformulation, immobilization, native -microbiota, source of carbon and energy

## Abstract

**Background:**

Biochar is a carbon-rich, porous material produced through controlled pyrolysis and widely applied in environmental and agricultural systems. It is increasingly used as a carrier material for microbial bioinoculant formulations and to stabilize anaerobic digestion processes by supporting microbial growth, colonization, and immobilization. However, the use of unsterilized biochar to reduce time, labor, and cost can lead to interference from native microbiota, potentially suppressing desired microbial strains and reducing process efficiency. Therefore, evaluating native microbial populations, growth-supporting ability, and immobilization potential is essential prior to its application.

**Methods:**

Six biochar samples obtained from the UK Biochar Research Center (UKBRC) were analyzed. Native and recoverable culturable microbial loads were enumerated for each biochar type. Growth-promoting potential was assessed using *E. coli* as a model organism. Additionally, microbial immobilization capacity was evaluated using both Gram-positive and Gram-negative bacterial strains. Scanning electron microscopy (SEM) was employed to further validate immobilization characteristics.

**Results:**

Distinct variations in native microbial loads were observed among the biochar samples, even under similar storage conditions. None of the biochars served as a carbon or energy source for *E. coli* ATCC 25922, but some delayed bacterial cell death, depending on the biochar type, indicating a possible viability preserving effect that warrants further investigation in formulated bioinoculant systems. Immobilization capacity varied significantly across biochars and was influenced by both the biomass source and pyrolysis temperature. All biochars showed statistically significant values (*P* < 0.05) for the immobilization of both the studied strains. SEM analysis confirmed differences in microbial attachment and surface characteristics.

**Conclusion:**

The study concludes that biochar potential to support growth and immobilization may vary according to production parameters and the type of biomass. Compatibility between biochar and microbial strains must be carefully evaluated before its use in bioinoculant formulations or other applications to ensure optimal performance and stability.

## 1 Introduction

Biochar, a carbonized biomass, is produced through the pyrolysis of organic biomass in an oxygen-limited environment (Shyam et al., 2025). It has emerged as a cornerstone of sustainable agriculture and circular bio-economy (Liu et al., 2021). Beyond its role in carbon sequestration, biochar serves as a porous carrier that stabilizes microbial activity (Ali et al., 2025; Köves et al., 2025). It enhances soil health and remediates contaminants by utilizing its surface area for pollutant adsorption while providing a habitat for microbes to drive biological degradation (Khan et al., 2024). Its efficacy as a carrier stems from its ability to provide a habitat for microbes, offering physical protection, enhancing the persistence of inoculants, and making the soil environment more productive, facilitating electron transfer and nutrient transformation (Palansooriya et al., 2019). The suitability of biochar as a carrier material for microbial formulations is not universal; rather, its quality is dictated by its pore architecture, pH, and nutrient chemistry, all of which are products of the chosen feedstock and the pyrolysis temperature (Ippolito et al., 2020). High-temperature biochars often exhibit increased surface area but may lose the surface functional groups that facilitate attachment (Antonangelo et al., 2019). Surface-bound contaminants, toxic ions, or an excessive sorption capacity can negatively impact the suitability of biochar as a carrier material, ultimately reducing microbial survival rates (Zhu et al., 2017). Specifically, biochar may harbor reactive organic compounds and heavy metals that are directly toxic to microorganisms; furthermore, it can adsorb signaling molecules, interrupting the formation of microbial biofilms on its surface (Zhang et al., 2023). Biochars derived from different biomass such as wood, husks, and shells using different temperatures, provide fundamentally different physical architectures, leading to significant variations in physicochemical nature, consequently available space for microbial colonization (Shen et al., 2018).

While the high temperatures of pyrolysis initially sterilize the material, biochar does not remain a blank slate (Bolan et al., 2023). During cooling, handling, and storage, the pores are rapidly colonized by indigenous microbes from the surrounding environment. This creates a complex feedback loop: the biochar changes the microbes, and the microbes change the biochar (Bolan et al., 2023). As specific microbes (such as N-fixers or P-cyclers) occupy these pores (Sharma et al., 2025), they secrete enzymes, such as urease and phosphatase, transforming biochar for nutrient processing (Bolan et al., 2023). However, a critical question remains: is the biochar providing these microbes with energy (carbon), or is it merely a structural refuge? On the biochar surface, immobilization is the key to maintaining microbial concentration and metabolic function under environmental stress (Li et al., 2022). This adhesion is highly dependent on the biological characteristics of the bacteria. Gram-positive bacteria, characterized by a thick peptidoglycan layer, often exhibit stronger adhesion to biochar surfaces (Wang et al., 2025; Zhang et al., 2022) while Gram-negative bacteria possess an outer membrane of lipopolysaccharides that creates different energetic barriers for attachment, relying more heavily on the production of Extracellular Polymeric Substances (EPS) to form stable biofilms (Wang et al., 2025).

Despite its potential, there is a lack of clarity regarding how different feedstocks, such as oakwood, softwood, and coconut, interact with varying bacterial cell wall structures. This study aims to quantify the indigenous bacterial load across diverse feedstocks to understand the pre-existing microbial landscape. Furthermore, it seeks to determine the metabolic role of biochar specifically, whether it serves as a metabolizable carbon source or primarily as a protective physical substrate. Finally, this research evaluates and compares the immobilization efficiency of wood-based and shell-based biochars using both Gram-positive and Gram-negative bacterial strains.

## 2 Materials and methods

### 2.1 Biochar preparation

**FIGURE 1:**
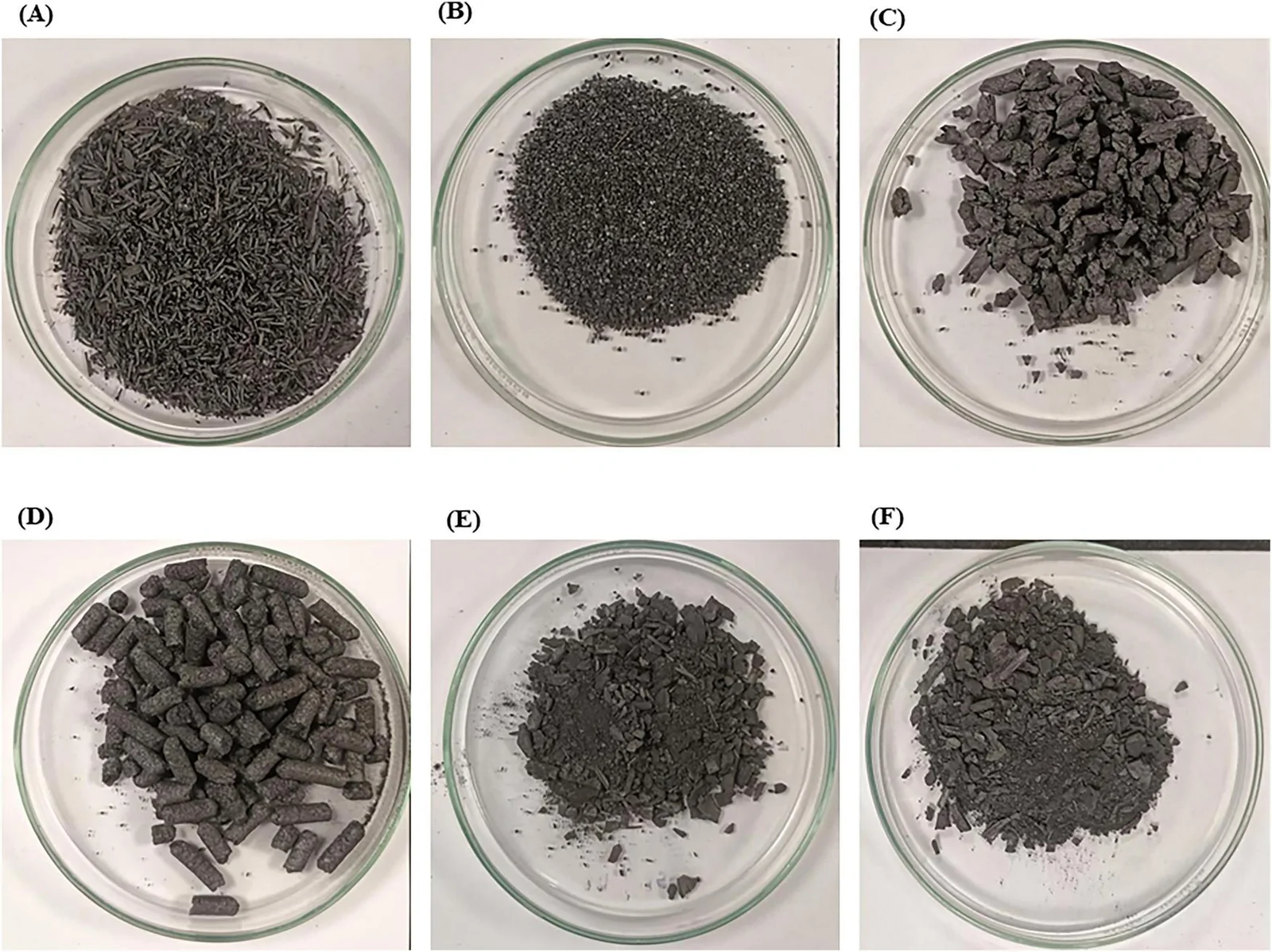
Morphological details of different types of biochar provided by UK Biochar Research Center (UKBRC) and used in the study. **(A)** Ricehusk, **(B)** Coconut shell, **(C)** Softwood700, **(D)** Softwood550, **(E)** Oakwood450, **(F)** Oakwood650.

Six different kinds of biochar (rice husk, coconut shell, softwood, and oakwood) was used in this study to understand their native microbial load, growth promotion potential (source of carbon and energy) and cell immobilization efficiency. The biochars except oakwood at 450 °C and oakwood at 650 °C used in this study was sourced from the UK Biochar Research Center (UKBRC) and produced using a pilot-scale rotary kiln pyrolysis reactor at various temperatures (softwood at 550 °C, softwood at 700 °C, and rice husk at 550 °C). In addition, two commercial biochars from oakwood at 450 °C and oakwood at 650 °C produced by Proininso (Malaga, Spain) via slow pyrolysis in a mono retort reactor from a bark-free holm oak wood residue, in the absence of oxygen. Coconut shell biochar was procured from Defiant Renewables Pvt Ltd, Pune (Maharashtra, India), which was prepared in a furnace at 500 °C, where the coconut shell was subjected to pyrolysis for 4 h. Biochars are shown in Figure 1. Biochar materials (rice husk, softwood and oakwood) were aseptically packed in a sterilized container and transported from the School of Chemical and Process Engineering, University of Leeds, Leeds, UK, by Dr. Andrew Ross at room temperature. The obtained biochar was stored in a closed container at room temperature for 2 days at the Symbiosis Center for Climate Change and Sustainability. Symbiosis International (Deemed University), Lavale, Pune-412115, India for growth and immobilization experiments. Physical and chemical properties and biochar characteristics are summarized in Table 1.

**TABLE 1:** Description of characteristic features of different kind of biochars used for the current study.

Properties	Units	Biochar types					
		SW700	SW550	RH	OW450	OW650	CS
Carbon (C_tot_)	wt% (d.b.)	90.21	85.52	48.69	65.70	76.50	69.68
Hydrogen (H)	wt% (d.b.)	1.83	2.77	1.24	2.70	1.40	2.37
Oxygen (O)	wt% (d.b.)	6.02	10.36	2.47	8.90	7.00	8.56
Molar H:C ratio	Molar ratio	0.24	0.39	0.28	0.40	0.20	0.41
Molar O:C ratio	Molar ratio	0.05	0.09	0.04	0.10	0.10	0.09
Ash content	wt% (d.b.)	1.89	1.25	47.93	11.70	14.30	0.88
pH	–	8.44	7.91	9.71	9.90	10.30	9.98
Moisture	wt% (a.r.)	1.00	1.52	1.54	29.30	15.20	4.16
Total Surface Area (BET)	m^2^/g	162.3	26.40	20.10	180.00	280.00	ND
Volatile Matter	wt% (d.b.)	6.60	14.20	7.48	21.10	11.80	22.07
Biochar C Stability	%	97.27	69.62	95.28	ND	ND	ND
Electrical conductivity	dS/m	0.16	0.09	0.48	0.63	0.45	ND

wt% (d.b.), weight percentage on dry basis; wt% (a.r.), weight percentage on an as-received basis, dS/m; deciSiemens per meter, SW700, Softwood700; SW550, Softwood550; RH, Ricehusk; OW450- Oakwood450; OW650, Oakwood 650; CS, Coconutshell; ND, No data; BET, Brunauer Emmett Teller analysis.

Source of Bacteria:

The bacterial isolates used in this study were sourced from various sources. The *Escherichia coli* (ATCC 25922) were obtained from Symbiosis Medical College for Women, Symbiosis International (Deemed University), Lavale, Pune. The pure culture of *Bacillus safensis* FO-36b was obtained from a project funded by DST-PURSE that is ongoing at SCCCS lab that was isolated from sludge reactor. The Gram-negative cocobacillus – *Pseudomonas fragi* strain 2T culture was obtained from another project at SCCCS lab that is funded by NMHS -MoEFCC isolated from soil near Tsomgo Lake in Sikkim.

### 2.2 Determination of bacterial load on biochar

To assess the native and recoverable bacterial load on the different types of biochar used in this study, 0.5 g of each biochar sample was aseptically collected and suspended in 4.5 mL of sterile normal saline (0.85% NaCl). The suspension was vortexed for 2 min to release the adhered bacterial cells from the biochar into the saline solution. The samples were then plated in two batches: the first batch was immediately serially diluted and plated using the spread plate method on R2A agar to enumerate the bacterial cells. The second batch was processed and plated after 24 h of incubation in saline with vortexing to assess recoverable culturable bacteria. All plates were incubated at 30 °C for 72 to 120 h, after which the visible colonies were enumerated and the results were calculated as colony-forming units (CFU) per gram of biochar (Figure 1).

### 2.3 Assessment of biochar as a source of carbon

To assess the growth promotion potential (source of carbon and energy) of selected biochar a defined minimal medium containing per liter of NaCl (100 mg), NH4Cl (100 mg), KH2PO4 (50 mg), KCl (10 mg), MgCl_2_⋅6H_2_O, CaCl_2_ (40 mg), NaHCO3 (2.5 mg), and a trace element solution (1 ml) as described by (Green et al., 2010) was supplemented with 20% (w/v) biochar as the sole carbon and energy source and sterilized by autoclaving. To understand the impact of the medium used on the growth of selected organisms, the same medium without biochar was used as a control. To provide a homogeneous surface area, all selected biochar was crushed using a pre-sterilized mortar and pestle, and the powder was passed through a 2.0 mm sieve. An overnight-grown 5% inoculum (OD 0.5 at 600 nm) of Escherichia coli ATCC 25922 was inoculated into experimental and control tubes in triplicate, and the tubes were incubated at 37 °C under aerobic conditions at 200 rpm in an incubator shaker with constant agitation to ensure uniform distribution of the biochar and nutrients. Growth was monitored at different time points (0, 12, 24, and 48 h) using CFU counts. Viable cell counts were determined via serial dilution and CFU analysis on agar plates, with all experiments performed in triplicate to ensure consistency.

### 2.4 Biochar immobilization and microbial quantification

The biochar immobilization experiment was conducted as discussed in Schommer et al. (2024). Briefly, to evaluate the bacterial cell immobilization capacity of the selected biochar materials, two different bacterial strains Gram-negative Psudomonas fragi strain 2T and Gram-positive Bacillus safensis FO-36b were selected and inoculated into Nutrient broth and incubated at 30 °C. After visible growth, (OD 0.5 at 600 nm), sterilized biochar (20 g/L concentration; autoclaved at 121 °C, 15 psi for 15 min) was added to the actively growing culture, and the tubes were again incubated at 30 °C and 100 rpm for 12 h. After incubation, the bacterial culture medium with biochar was filtered using sterile filter paper to separate the biochar, which was subsequently washed with sterile distilled water to remove loosely attached bacterial cells (Schommer et al., 2024). To enumerate the number of immobilized cells, the Total Viable Count (TVC) was performed. For that the immobilized biochar was suspended in sterile phosphate-buffered saline (PBS) and vortexed for 2 min to detach the cells, followed by the preparation of serial dilution series using a standard protocol. The drop plate method, as discussed in (Reed and Reed, 1948) and optimized in our laboratory, was used for the CFU count. To calculate CFUg^–1^ following formula was used:


C⁢F⁢U/g=M⁢e⁢a⁢n⁢c⁢o⁢l⁢o⁢n⁢i⁢e⁢s⁢p⁢e⁢r⁢d⁢r⁢o⁢p/D⁢r⁢o⁢p⁢v⁢o⁢l⁢u⁢m⁢e⁢i⁢n

m⁢L*D⁢i⁢l⁢u⁢t⁢i⁢o⁢n⁢F⁢a⁢c⁢t⁢o⁢r

### 2.5 Scanning electron microscopy of cell-immobilized biochars

Scanning electron microscopy (SEM) of the cell-immobilized biochar was performed to qualitatively confirm microbial attachment and analyze the spatial distribution of bacterial cells across the biochar’s surface morphology. Samples were prepared for SEM using chemical fixation followed by critical-point drying (CPD). Initially, specimens were fixed in 2% glutaraldehyde and stored at 4 °C. Prior to imaging, the samples underwent a series of washes: three times with Phosphate-Buffered Saline (PBS) and twice with distilled water (D/W). Dehydration was performed using a graded ethanol series of 30%, 50%, 70%, and 95%, incubating for 15 min at each concentration. The final dehydration step in 100% ethanol was repeated three times. Following dehydration and CPD, the samples were sputter-coated with a 10–15 nm layer of gold or gold-palladium to enhance conductivity. Imaging was performed using a Zeiss EVO MA-15 scanning electron microscope at the Agharkar Research Institute (ARI). The samples were observed at accelerating voltages of 2.0–3.0 kV, with a working distance (WD) of 8 mm and a 30 μm aperture. Micrographs were captured at magnifications ranging from 5,000× for surface surveys to 20,000× for detailed observation of cellular attachment.

### 2.6 Statistical analysis

To compare how effectively the six different biochars immobilized the two bacterial strains (*P. fragi* and *B. safensis*), Two-way ANOVA with Replication was performed using the XLMiner Analysis ToolPak with a significance threshold of α = 0.05. Mean colony counts (CFUg^–1^) were used directly for analysis. Replication across three plates per treatment ensured robustness of the comparison.

To investigate the relationship between biochar physicochemical properties and immobilization outcomes, Spearman rank correlation analysis was performed between five properties from Supplementary Table 1 (pH, BET surface area, total carbon, ash content, and volatile matter) and the log10-transformed immobilization densities of both *P. fragi* strain 2T and *B. safensis* FO-36b. CFUg^–1^ values were converted to log10 prior to analysis normalize the exponential nature of bacterial counts. Coconut shell biochar was excluded from BET surface area correlations due to unavailable data (ND), reducing the sample size to *n* = 5 for those comparisons; all other correlations were conducted with *n* = 6. Rank differences (d) were calculated for each biochar pair, and the Spearman rho (ρ) was computed using the formula ρ = 1 - (6Σd^2^/n(n^2^-1)). Statistical significance was determined using a two-tailed t-distribution with (n-2) degrees of freedom at α = 0.05.

## 3 Results

### 3.1 Bacterial load on biochar

**FIGURE 2:**
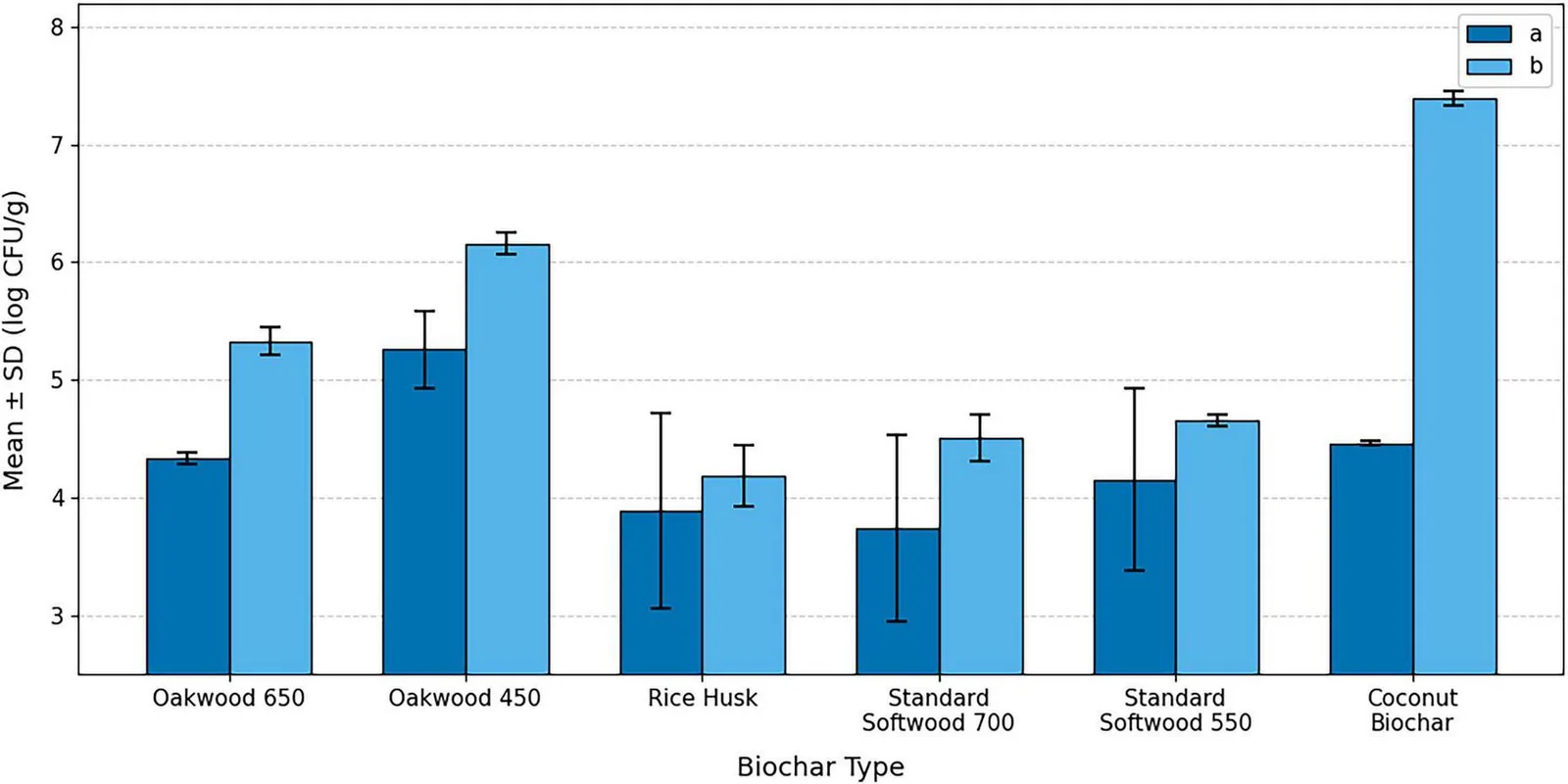
Graph represents the bacterial load on different biochar types enumerated at two different time points. (a) Is when the samples were plated immediately after suspension and vortexing (T0) and (b) data taken after 24 h of incubation (T24).

Microbial load on the biochar samples was assessed at two time points: immediately after suspension (T0) and after a 24-h incubation period, to capture recoverable culturable bacteria (T24), as shown in Figure 2. Upon immediate plating (Batch 1), all biochar samples were found to harbor indigenous microbial populations, though the abundance varied significantly by feedstock and production temperature. Standard Softwood 700 biochar contained the lowest initial bacterial load at 5.4 × 10^3^CFU g^–1^. In contrast, Oakwood 450 biochar exhibited a markedly higher initial load of 1.8 × 10^5^ CFU g^–1^, indicating that lower pyrolysis temperatures and wood types may preserve more native microbes. After 24 h of incubation in saline solution (Batch 2), the recoverable culturable bacterial counts increased across all samples, but the magnitude of growth differed drastically between feedstocks, rice husk biochar exhibited the most stable profile, with the load increasing modestly from 7.7 × 10^3^ CFU g^–1^ to 2 × 10^4^ CFU g^–1^. Whereas, coconut Shell Biochar demonstrated a massive surge in microbial activity, reaching 2.6 × 10^7^ CFU g^–1^ from an initial 2.9 × 10^4^ CFU g^–1^. This represents a growth of nearly three orders of magnitude, the highest recorded in the study.

### 3.2 Biochar as a source of carbon

**FIGURE 3:**
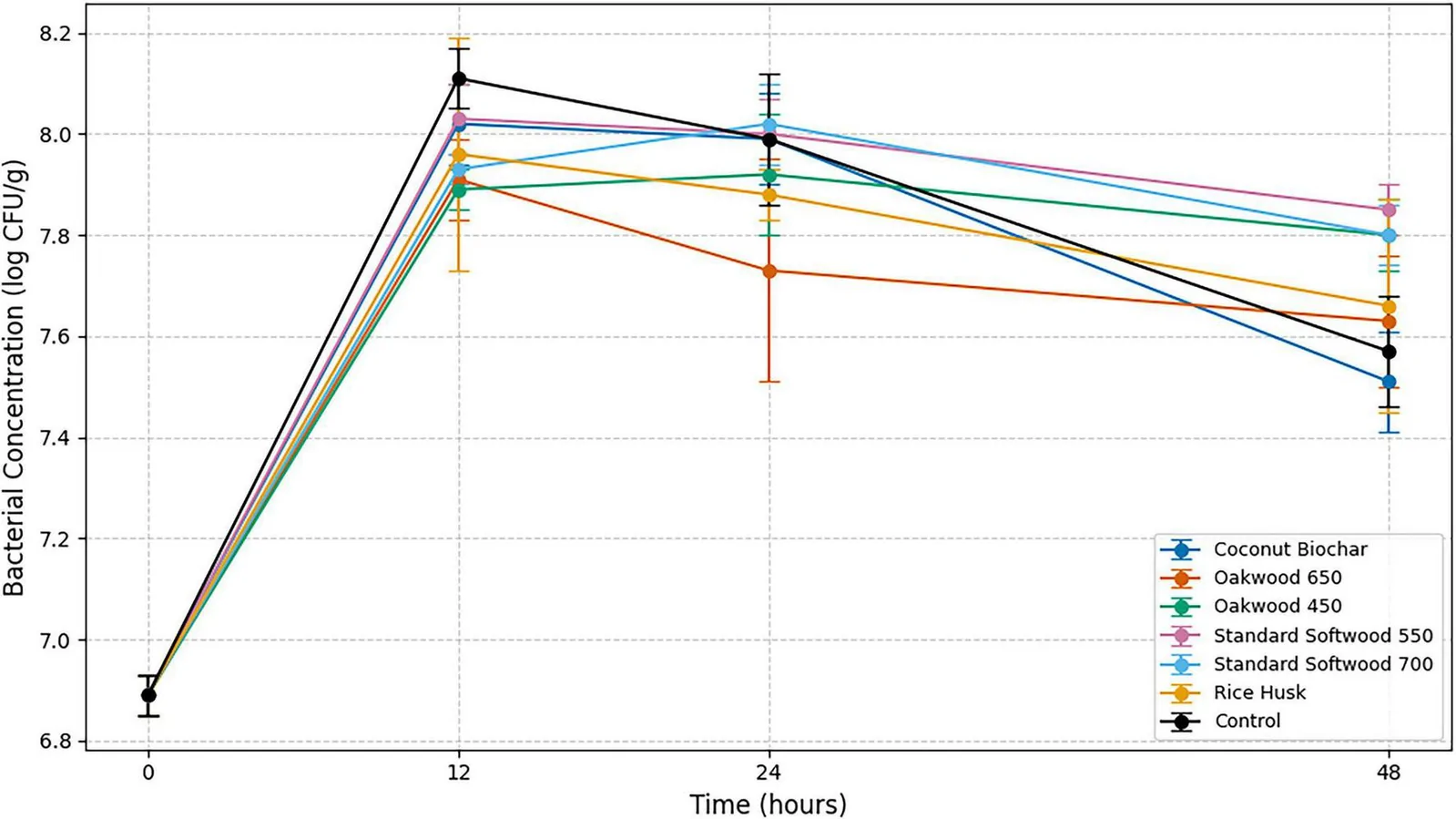
Comparative analysis of bacterial growth patterns in minimal medium supplemented with and without Biochar.

The growth experiment showed that after inoculation, bacterial counts increased for up to 12 h in both the biochar-amended and the non-biochar-amended minimal medium. At 12 h, the cell count was highest in minimal medium without biochar, while the biochar-amended medium showed variable growth, better in softwood 550. Softwood 700 showed less growth than softwood at 550, but by 24 h, it was almost the same. Lowest cell counts at 12 h were observed in both oakwood biochars, but oakwood 650 showed an immediate decline after 12 h, followed by a steady decline after 24 h. In comparison, oakwood 450 maintained longer cellular viability, showing steady growth even after 12 h and declined at a steady rate after 24 h. Coconut shell biochar and medium without biochar showed quicker death of inoculated cells after 24 h. The observed reduction in bacterial counts over time indicates that biochar does not serve as a metabolizable carbon source. However, the comparatively sharper decline in the minimal medium without biochar suggests that biochar may enhance bacterial persistence by providing a protective microenvironment rather than acting as a nutrient substrate (Figure 3).

### 3.3 Biochar immobilization and microbial quantification

**FIGURE 4:**
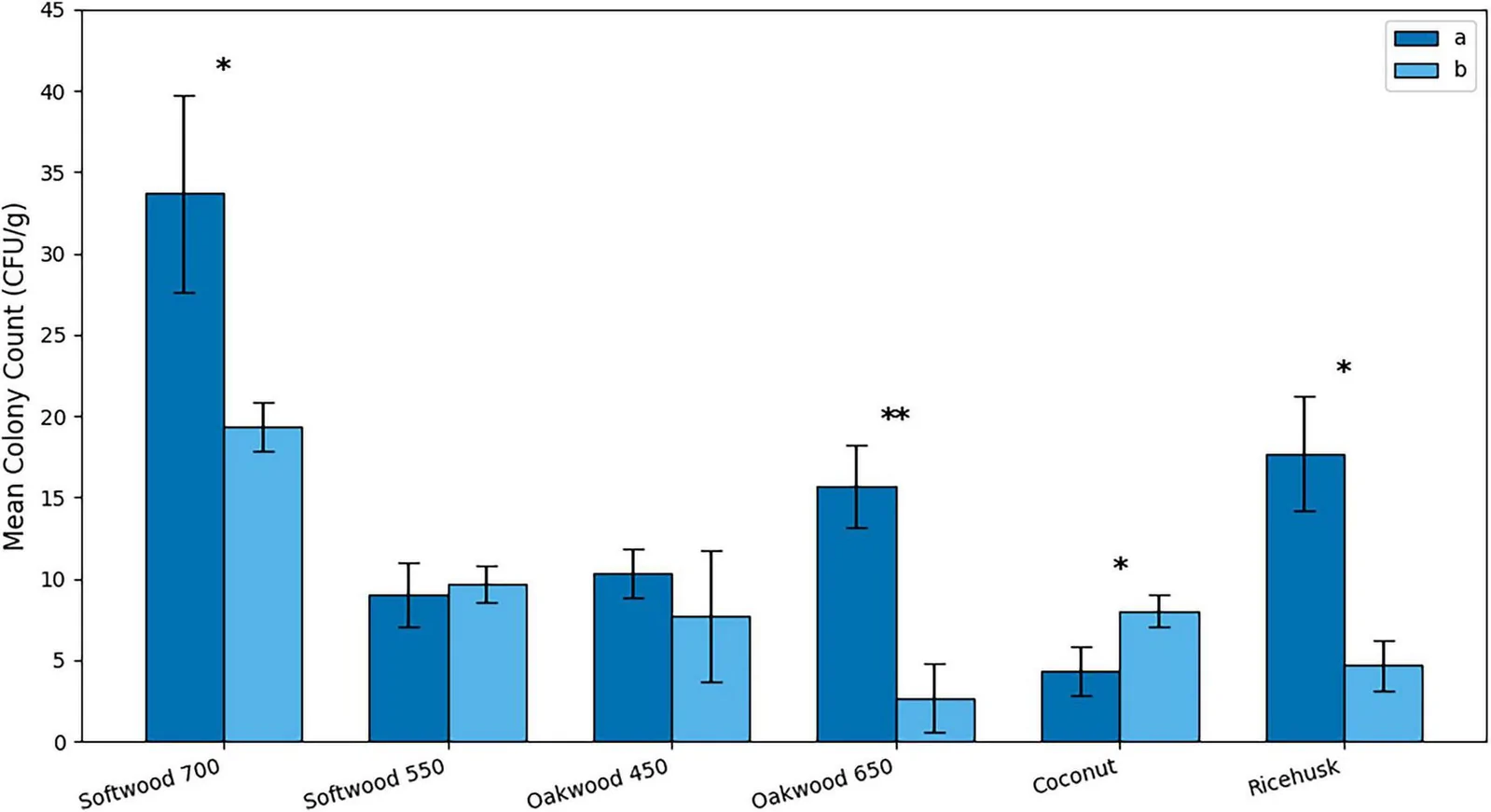
Comparison of immobilization capacities of six biochar substrates for (a) Gram-negative strain *P. fragi* strain 2T and (b) *B. safensis* FO-36b, where *P < 0.05 Statistically Significant difference between organisms; **P < 0.01 Highly Significant difference between organisms; (no star) *P* > 0.05 Not Significant (ns).

This study demonstrates that biochar type significantly influences the immobilization capacity of the two tested strains, although contrary to general expectations, the Gram-negative *P. fragi* strain 2T frequently achieved higher peak concentrations than the Gram-positive *B. safensis* FO-36b across several substrates. Softwood 700 emerged as a top-performing carrier for both strains, supporting the highest immobilization for *P. fragi* strain 2T (1.6 × 10^7^ CFU g^–1^) and a strong presence for *B. safensis* FO-36b (1.9 × 10^5^ CFUg^–1^). Interestingly, a sharp disparity in efficiency was noted on softwood 550; while it supported *B. safensis* FO-36b at 9.6 × 10^4^ CFUg^–1^, *P. fragi* strain 2T showed recorded CFU of 4.5 × 10^5^ CFUg^–1^. For *B. safensis* FO-36b, the highest density was observed on coconut biochar (8.0 × 10^6^ CFUg^–1^) and Oakwood 450 (7.6 × 10^6^ CFUg^–1^), suggesting that these materials provide a highly conducive surface environment for this strain. Conversely, while rice husk performed well for *P. fragi* strain 2T (8.8 × 10^6^ CFUg^–1^), it showed a lower immobilization potential for *B. safensis* FO-36b (4.6 × 10^6^ CFUg^–1^), indicating that substrate suitability is highly strain-dependent (Figure 4).

### 3.4 Scanning electron micrograph analysis

**FIGURE 5:**
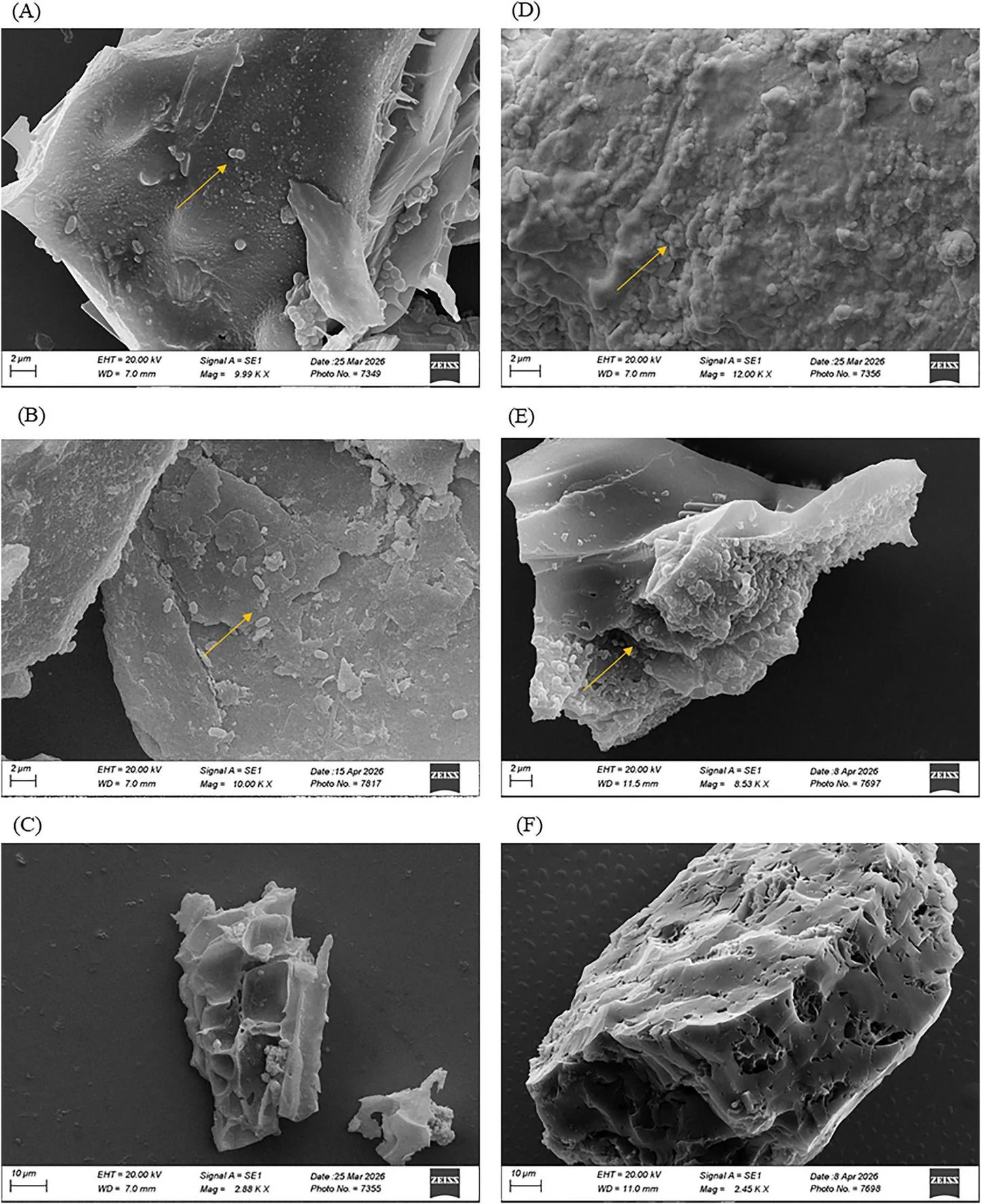
Scanning electron micrograph (SEM) of immobilized cells in biochars. **(A)** Ricehusk *P. fragi* strain 2T, **(B)** Ricehusk *B. safensis* FO-36b, **(C)** Ricehusk control, **(D)** Coconut *P. fragi* strain 2T, **(E)** Coconut *B. safensis* FO-36b, **(F)** Coconut control.

**FIGURE 6:**
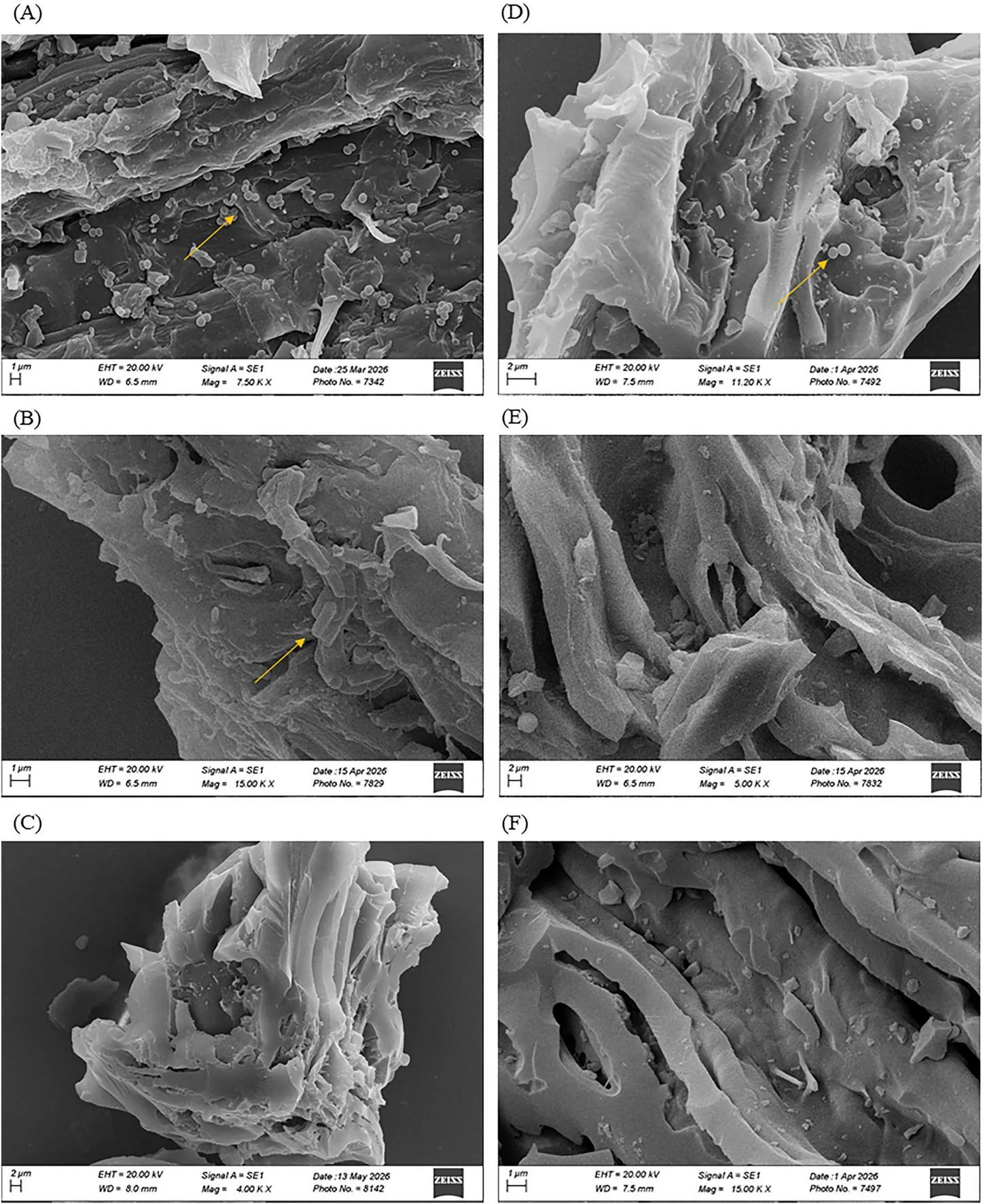
Scanning electron micrograph (SEM) of immobilized cells in biochars. **(A)** Softwood550 *P. fragi* strain 2T, **(B)** Softwood550 *B. safensis* FO-36b **(C)** Softwood550 control **(D)** Softwood700 *P. fragi* strain 2T, **(E)** Softwood700 *B. safensis* FO-36b **(F)** Softwood700 control.

**FIGURE 7:**
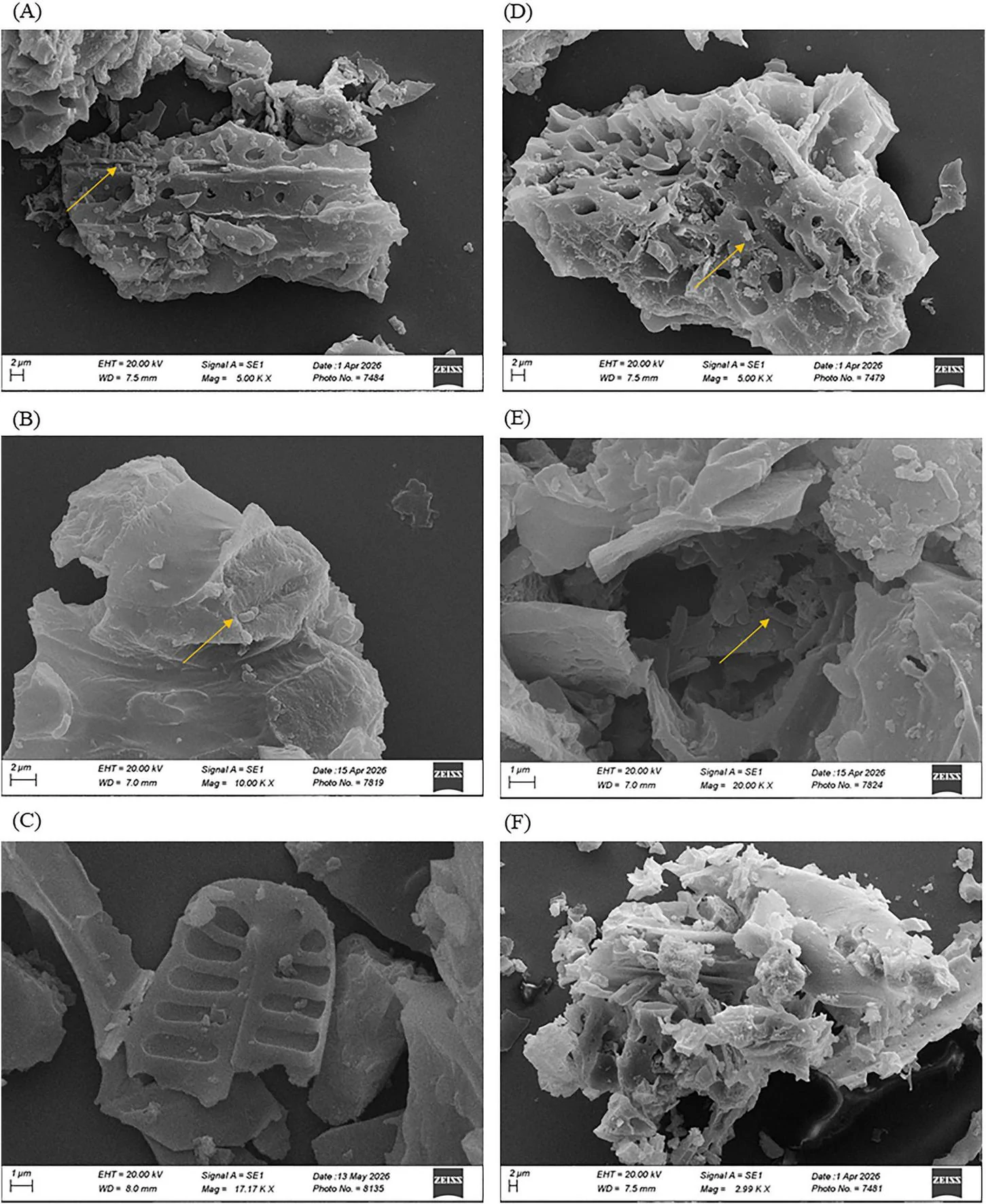
Scanning electron micrograph (SEM) of immobilized cells in biochars. **(A)** Oakwood450 *P. fragi* strain 2T. **(B)** Oakwood450 *B. safensis* FO-36b, **(C)** Oakwood450 control, **(D)** Oakwood650 *P. fragi* strain 2T, **(E)** Oakwood650 *B. safensis* FO-36b, **(F)** Oakwood650 control.

The SEM analysis revealed distinct structural variations among the biochar types and the successful immobilization of the bacterial strains. SEM images (micrographs) of softwood 550 and 700, and oakwood 450 and 650 substrates revealed a highly porous, honeycombed architecture with long channels, whereas the rice husk and coconut biochars exhibited more irregular surface textures. In the control samples (un-immobilized biochar), surfaces remained clean, with no visible bacterial cells, whereas the inoculated samples (*P. fragi* strain 2T and *B. safensis* FO-36b) clearly showed bacterial cells colonizing the substrates. Micrographs of (*P. fragi* strain 2T) and (*B. safensis* FO-36b) revealed distinct morphological differences. *P. fragi* strain 2T appeared as coccus-shaped, while *B. safensis* FO-36b presented as slightly more elongated bacilli. These cells were observed adhering to the outer surfaces and nesting within the pores of biochar, forming small clusters. This visual evidence shows that the biochar’s internal pore structure provides an effective protective environment for microbial attachment and colonization (Figures 5, 6, 7).

### 3.5 Statistical analysis

The two-way ANOVA revealed differences in immobilization performance. It was observed that *P. fragi* strain 2T achieved higher overall immobilization density as compared with *B safensis* FO-36b (F_1,24_ = 49.11, *P* < 0.001) (Figure 4), Furthermore, the analysis confirmed that the Biochar feedstock significantly influenced total immobilization capacity (F_5,24_ = 42.42, *P* < 0.001). Regarding specific substrates, *P. fragi* strain 2T exhibited significantly higher growth on Softwood 700 *P* < 0.05 and Oakwood 650 *P* < 0.01 and rice husk *P* < 0.05. *B. safensis* FO-36b showed significant values for coconut biochar, *P* < 0.05, which was the only material that favored *Bacillus*. While softwood 550 and oakwood 450 supported both organisms, they did so at lower overall density, with no statistically significant difference between the two strains (*P* > 0.05; Figure 4).

Spearman rank correlation analysis between five biochar physicochemical properties (pH, BET surface area, total carbon, ash content, and volatile matter) and immobilization outcomes revealed that volatile matter content was the only property to show a statistically significant correlation, positively associating with *B. safensis* FO-36b immobilization density (ρ = +0.829, *p* = 0.04, Supplementary Table 1). No significant correlations were identified between the remaining properties and immobilization outcomes for either strain (*p* > 0.05).

## 4 Discussion

Due to superior carbon storage capacity and several other advantages, biochar is generally recommended for sustainable agriculture and environmental practices (Mirzaei et al., 2026). It is frequently used to stabilize processes and as a carrier material for immobilization of microbes for biopesticides, biofertilizers, and other industrial applications. It is generally observed that 60–80% of the carbon in biochar is recalcitrant, with only a limited fraction (2–10%) being labile, which supports little to no microbial growth during storage (Chagas et al., 2022). It was generally found that, even after prolonged storage at room temperature, no visible bacterial or fungal growth was observed on the biochar. In light of the above observation, non-sterilized biochar is sometimes used in microbial formulations or for process stabilization to save time and cost (Kanishka et al., 2025). But during application or formulation, the native bacterial and fungal cells present on biochar surface or inside the pore can grow and suppress the growth of desired microorganisms inoculated from outside, thereby reducing the formulation’s efficacy and shelf life. The colonization efficiency of microbes on different biochars depends on biochar chemistry, which is subsequently controlled by the type of biomass and pyrolysis conditions used during biochar formation. On similar line, the immobilization potential of bacterial cells on biochar surface depends on the type of biochar, the pyrolysis process, and cell surface adhesin properties of selected bacterial cell. The same cell may behave differently in terms of viability and growth on different biochars depending on biochar chemistry. Therefore, the analysis of the native microbial population of biochar, its growth-promoting potential, and its cell-immobilization capacity should be determined on a case-by-case basis. The objective of the current study was to investigate the native bacterial load on different types of biochar, their growth promotion potential (source of carbon and energy), and microbial immobilization capacity.

### 4.1 Native microbial load on different biochar

The results indicate a substantial increase in bacterial cell count after 24 h of incubation (T24) in comparison to cell count obtained just after inoculation and vortexing (T0). The increase in viable cell count after 24 h is likely due to utilization of residual carbon released by biochar materials in suspending matrix. This extended 24-h incubation period provides sufficient time for the physiological recovery and growth of microbes present on the surface and within the pores, while simultaneously facilitating the stress recovery and gradual detachment of cells that were previously sequestered deep within the biochar’s complex internal architecture and inaccessible during the initial 2-min vortexing (Tavares et al., 2020; Tu et al., 2020). The higher cell count observed in coconut shell biochar, in comparison to other suggests a superior synergy between its physical structure and chemical composition. It might be due to that coconut shell biochar provides more labile carbon, less toxicity and effective adhesion within its pores and on its surface. These traits, combined with high water-holding capacity and surface area (Gryta et al., 2023), enable indigenous bacteria to survive osmotic stress, utilize residual nutrients within the matrix, and remain shielded in protective niches (Zhang et al., 2024). Consequently, over the 24-h window, these populations can undergo cell division and detach into the saline, resulting in significantly higher recovery of culturable cells than those plated immediately after vortexing. Thus, our result indicates that all types of biochar used in this study support native bacterial growth which can override the growth of non-indigenous microbes inoculated for formulation. Therefore, using autoclaving or other methods of sterilization would be an effective option for increasing the efficacy, shelf life of products, and process stability.

### 4.2 Biochar as carbon source vs. protective habitat

Results of experiments conducted to understand biochar as a source of carbon and energy showed initial (12 h) growth in with and without biochar-amended medium after 12 h of incubation. It suggests that inoculated cells utilized residual or carryover carbon present as contaminants in minimal medium and showed initial growth. We also found that the cell count was slightly higher in minimal medium alone used as control than minimal medium supplemented with biochar. This finding was consistent with all kinds of biochar used in our experiment (Figure 3), which confirms that the growth of inoculated *E. coli* ATCC 25922 cells was due to residual carbon present in minimal medium and not due to the biochar. After that, the minimal medium with and without biochar showed a decline in *E*. *coli* count, indicating carbon limitation and cell death. This can be linked to the generalized view that most biochar carbon is recalcitrant and can only support the growth of fast-growing heterotrophs (Joseph et al., 2021; Xu et al., 2021). The study also showed that a slower decline in cell count in the biochar-amended medium may be due to the refugium effect, which shields cells from stress and improves the primary conditions of biochar, such as pH, moisture, and adsorption capacity for toxins. The overall study suggests that biochar structures can enhance microbial persistence rather than providing nutrients for their growth (Joseph et al., 2021; Xu et al., 2021; Zhao et al., 2024). In this study, we used *E. coli cells*, but the growth-supporting potential depends on the type of biochar and the nature of microbial cells. For some other taxa, same biochar type can give different results. Thus, the growth promotion, suppression and refugium effect could be different with different biochar and bacterial cell combinations and should be tested before use them for any formulation

### 4.3 Biochar as a substrate for bacterial immobilization

The findings of this study demonstrate that biochar type and production temperature significantly influence immobilization capacity, though the results are highly strain-dependent. Softwood 700 showed the highest cell counts for *P. fragi* (1.6 * 10^7^ CFUg^–1^) and results for *B. safensis*, suggesting that high-temperature pyrolysis of softwood creates a stable, high-surface-area environment with accessible pore structures conducive to both strains (Wang et al., 2025). In contrast Softwood 550 supported *B. safensis* but suppress the growth *P. fragi* (4.5 × 10^5^ CFUg^–1^), showing how minor variations in pyrolysis temperature can alter surface chemistry and pore accessibility enough to impact strain-level compatibility (Li et al., 2025). While oakwood 450 and coconut biochars provided the most favorable environments for *B. safensis* (7.6 × 10^6^ and 8.0 × 10^6^ CFUg^–1^, respectively), Rice husk showed a clear strain-substrate preference, performing well for *P. fragi* but showing the lowest potential for *B. safensis*. These observations confirm that biochar is a promising substitute for traditional carriers, though its suitability depends on pore size, pH, and surface functionality (Li et al., 2023). Contrary to the general expectation that Gram-positive bacteria typically colonize biochar more effectively due to their thicker peptidoglycan layer (Geng et al., 2022), in our study the Gram-negative *P. fragi* Strain 2T showed better immobilization than Gram positive *B. safensis*. The bacterial immobilization on sterilized biochar materials depends on several factors, including physicochemical features of biochar like pH, surface structure, pore size surface area and many more other. In addition to biochar features, the bacterial cell properties like exopolysaccharide production, biofilm formation, cell size, nature of cell surface charges, adhesion potential, incubation time, cellular compatibility with biochar type etc. Therefore, the immobilization potential of biochar can vary with physicochical nature and type of microbial cells and advice to study separately for each taxa and biochar type.

### 4.4 Comparative evaluation of biochar as a carbon source and microbial immobilization carrier

Although experiments on carbon sources and immobilization were separate, analyzing them together better evaluates each biochar’s suitability for microbial uses. None of the tested biochars supported growth in carbon-deficient minimal medium, indicating the carbon of tested biochar is likely resistant to degradation and not easily metabolized by *E. coli*. However, viability in cell death rate among the tested biochar types, suggested difference in survival rate of same kind of cells on various biochars (Xiong et al., 2026) and a matter of further investigation with different microbial taxa.

Although the coconut-based biochar showed rapid decline in bacterial viability during the carbon source experiment using E. coli, while it achieved the highest immobilization efficiency for *B. safensis in our immobilization experiment*. Furthermore, Rice husk biochar showed modest survival of tested cells *in carbon source experiments* but represents high immobilization of Gram-negative strain *P. fragi strain 2T*. Thus, supporting our hypothesis that growth promotion potential and immobilization are depends on physicochemical features of biochar and tested bacterial taxa used.

When compared with immobilization outcomes, these findings show significant differences in the multifunctional capabilities of the biochars. Softwood 550 extended bacterial survival better than most biochars in the carbon source experiment but showed lower immobilization densities, especially for strain *P. fragi*. By contrast, Softwood 700 achieved the highest immobilization efficiency for Gram-negative strain *P. fragi* and significant colonization by *Bacillus safensis*, despite only moderately enhancing persistence under carbon-limited conditions. Thus, traits promoting attachment may not necessarily support survival during nutrient scarcity.

Oakwood biochars highlight this distinction. Oakwood 450 sustained bacterial viability longer than Oakwood 650 in the carbon source experiment and supported high immobilization densities for *B. safensis*. In contrast, Oakwood 650 showed a quicker survival decline but better immobilization of strain *P. fragi*. These responses show pyrolysis temperature shapes surface chemistry, pore structure, and suitability for microbial survival or attachment (Wang et al., 2025).

The impact of pyrolysis temperature on immobilization was particularly seen in the softwood biochars, where higher temperatures supported better colonization of both type of selected microorganism. Temperature-induced changes in surface area, porosity, and chemistry can make higher-temperature biochar as more efficient microbial carriers (Wang et al., 2025). However, in the case of oakwood, a contrasting response was observed. Oakwood 450 (low temperature) was more efficient for the Gram-positive *B. safensis*, whereas oakwood 650 (High temperature) give better support to Gram-negative *P. fragi*. This may be because Gram-positive strains often benefit from the more available nutrients, organic substrates, and milder pH levels associated with lower-temperature chars (Kracmarova-Farren et al., 2024; Shabir et al., 2024). Alternatively, Gram-negative bacteria may be better equipped to exploit the higher surface area, more developed micro- mesopores, and alkaline or hydrophobic surface characteristics of high-temperature biochars (Vaiškûnaitë et al., 2024; Zhao et al., 2020).

## 5 Conclusion

Considering the importance of biochar in agricultural, environmental, and industrial applications and their use as carrier material for microbial-based bioinoculant formulations current study focused on understanding of native bacterial load of different biochar types, their growth-promoting potential, and their ability to support bacterial cell immobilization. Our results indicate that although the biochar generation is based on high-temperature pyrolysis but after storage different biochar types harbor diverse bacterial loads, which increase with further incubation and compromise the initial sterility of biochar. Furthermore, our data also indicates that none of the selected biochars supported the growth of the *E. coli* ATCC 25922 strains used in the current study. Results of immobilization study indicate that the immobilization potentials of bacterial cells depend on physicochemical traits of biochar and nature of bacterial taxa used for immobilization. Thus, the growth-promoting and immobilization potential of biochar depends on the type of microbial taxa selected for study and on the nature of the available labile carbon and other compounds produced on the biochar surface during its production. Even biochar produced from the same biomass using different pyrolysis methods exhibits different behaviors. Thus, in conclusion, a compatibility analysis of biochar type and selected microbial taxa is essential before using it for any formulation or process stabilization. Sterilization may be preferable when the goal is to minimize competition from culturable native bacteria, but the effect of sterilization on biochar properties and formulation performance should be evaluated for each application.

## 6 Limitations of the study

Following are the limitations of this study:

1. Only aerobic culturable bacteria were enumerated using R2A agar, thereby omitting non-culturable, viable but non-culturable (VBNC), and fungal fractions.
2. The total community composition was not determined.
3. Carbon mineralization, dissolved organic carbon (DOC), and CO2 dynamics were not measured. Consequently, future research incorporating 16S rRNA amplicon sequencing, shotgun metagenomics, and carbon dynamic assays is recommended to fully characterize the native microbial ecology and chemical stability of stored biochar across diverse feedstocks.
4. Future studies could include natural and artificial carriers for microbial immobilization.
5. Future experiment on using biochar as carbon source we can consider representative strains from multiple taxa to get a broader understanding of the concept.

## Data Availability

The original contributions presented in the study are included in the article/Supplementary material, further inquiries can be directed to the corresponding author.
