# Supplementary material for: Microbial load, carbon utilization potential, and immobilization efficiency vary across biochar types: implications for bioinoculant formulation
Source: Front Microbiol. 2026 Sep 1;17:1869658. doi: 10.3389/fmicb.2026.1869658 (PMC13574953; doi:10.3389/fmicb.2026.1869658)
Supplement: Supplementary file 1 [file Table_1.docx]

**Supplementary Data**

**Supplementary Table 1: Spearman Correlation with biochar properties**

| Property | PF  (ρ) | PF  (p-value) | BS  (ρ) | BS  (p-value) | BS  (Sig) |
| --- | --- | --- | --- | --- | --- |
| BET Surface Area+ | 0.3 | 0.62 | 0.6 | 0.28 | ns |
| pH | 0.03 | 0.95 | 0.6 | 0.21 | ns |
| Total Carbon | 0.03 | 0.95 | −0.78 | 0.07 | ns |
| Ash Content | 0.43 | 0.39 | 0.43 | 0.39 | ns |
| Volatile Matter | −0.65 | 0.16 | **0.829** | **0.04** | ***** |

PF; *Pseudomonas fragi* strain 2T*,* BS; *Bacillus safensis* FO-36b*, Sig;Significance,* ρ- Spearman Rho

+ Coconut Shell biochar excluded from BET correlations due to unavailable data (ND).
ns = not significant (p > 0.05); * = significant (p < 0.05)
